# *RbohA* coordinates lateral root emergence in common bean

**DOI:** 10.1080/19420889.2018.1467188

**Published:** 2018-05-11

**Authors:** Manoj-Kumar Arthikala, Carmen Quinto

**Affiliations:** a Ciencias Agrogenómicas, Escuela Nacional de Estudios Superiores Unidad León, Universidad Nacional Autónoma de México (UNAM), León, Guanajuato, México; b Departamento de Biología Molecular de Plantas, Instituto de Biotecnología, Universidad Nacional Autónoma de México (UNAM), Cuernavaca, Morelos, México

**Keywords:** common bean, lateral root emergence, NADPH oxidase, promoter *RbohA*, *RbohA-RNAi*

## Abstract

Respiratory burst oxidase homologs (RBOHs) constitute a multigene family in plants. These reactive oxygen species (ROS)-generating enzymes participate in diverse biological processes. We previously demonstrated that *PvRbohB* plays an important role in lateral root (LR) development in *Phaseolus vulgaris*. However, little is known about the roles of other *Rboh* members in root development. Here, we report that *PvRbohA* is involved in LR emergence in *P. vulgaris*. *PvRbohA* was expressed in wild-type *P. vulgaris* root tissues, e.g., the radicle, inter LR zone, and LR zone, and its expression increased during LR formation. Analysis of the spatio-temporal expression patterns of a reporter construct under the control of the *PvRbohA* promoter (*PvRbohA*::GUS-GFP) in transgenic roots showed that *PvRbohA* was active at all three stages of LR development, but its spatial expression pattern varied at each stage. The relative expression levels of *PvRbohA* during LR formation correlated with the activity of *PvRbohA* promoter. Furthermore, upon *PvRbohA* transcript silencing, LR growth was significantly altered in transgenic hairy roots. These findings suggest that *RbohA* participates in LR initiation, emergence, and development in the legume *P. vulgaris* by delimiting the region for LR emergence.

Root branching through lateral root (LR) formation is an important element in the adaptability of the plant root system to its environment. The degree of root branching affects the efficiency of water uptake, nutrient acquisition, and anchoring of the plant in the soil. Molecular genetics and physiological studies in *Arabidopsis thaliana* have demonstrated that LR formation is dependent on auxin, which regulates most steps of LR development, such as LR founder cell specification, LR initiation, LR primordium development, and LR emergence [1–4]. Reactive oxygen species (ROS) derived from the activity of NADPH oxidases are thought to function as important signals during auxin-regulated LR formation, as respiratory burst oxidase homolog (RBOH)-mediated ROS production facilitates LR emergence in *Arabidopsis* [5]. There is compelling evidence that ROS derived from NADPH oxidase have important roles during adventitious root formation [6] and root-to-shoot communication [7]. Among ROS, superoxide anion, hydrogen peroxide and hydroxyl radical are involved in cell wall modifications during numerous plant developmental processes, including root hair development [8,9]. ROS production in extracellular spaces depends on several classes of enzymes, including RBOH, whose activity is crucial. Treatment with the RBOH inhibitor diphenyleneiodonium (DPI) reduces the meristem cell number in *Arabidopsis* primary roots [10]. Accordingly, superoxide anions primarily accumulate in the meristematic region of *Arabidopsis*, and DPI treatment has a negative effect on ROS production and primary root growth [10]. Furthermore, loss-of-function of *AtRBOHC* and *AtRBOHF* significantly affects primary root growth [11]. Recent studies have demonstrated that disrupting (or enhancing) expression of RBOH in LR primordia and/or overlying root tissues decelerates (or accelerates) the development and emergence of LRs [5]. In *Phaseolus*, silencing of *PvRbohB* resulted in a significant reduction in ROS levels and LR density [12,13].

It is important to examine the roles of different RBOH gene family members, as each member can play distinct roles in the same biological process, ranging from synergistic to non-redundant functions. Therefore, in the current study, we downregulated *PvRbohA* expression via RNAi-mediated gene silencing and analyzed root growth parameters. We also assessed the spatio-temporal activity of the *PvRbohA* promoter during LR emergence in common bean (*Phaseolus vulgaris*).

## PvRbohA expression in P. vulgaris roots

**Figure 1.**
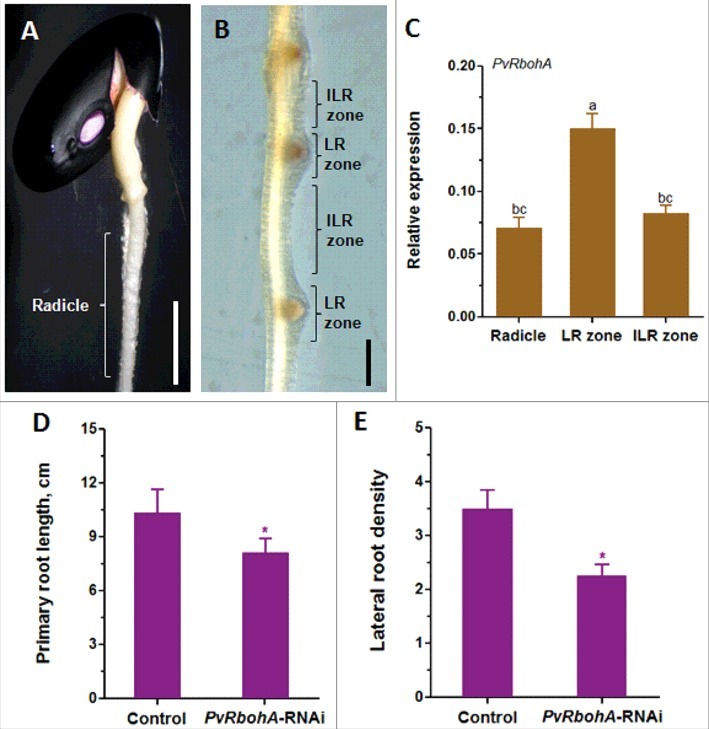
Quantitative RT-PCR analysis of *PvRbohA* expression and root growth parameters in *Phaseolus vulgaris*. A representative wild-type *P. vulgaris* germinating seedling showing (A) a radicle (from 2-day-old seedling) and (B) ILR and LR zones of the root (from 4-day-old seedling). (C) RT-qPCR expression analysis of *PvRbohA* from mRNA isolated from radicles, ILR zones, and LR zones of wild-type *P. vulgaris* seedlings. Transcript accumulation was normalized to the expression of the *Ef1α* and *IDE* reference genes. The statistical significance of differences between the different root zones was calculated by ANOVA and Tukey's Multiple Comparison Test, where different letters indicate significance differences (*P* < 0.001). *P. vulgaris* composite plants containing transgenic hairy roots were analyzed at 10 days post transplantation. (D) Primary root length and (E) lateral root density in control and *PvRbohA*-*RNAi* roots. The statistical significance of the differences between control and RNAi root samples was determined using an unpaired two-tailed Student's *t*-test (*P<0.05). The reported values represent three biological replicates (C; *n* > 9, D, E; n > 21). Error bars represent the means ± SEM. Scale bar: A, 5 mm; B, 2 mm. ILR zone, inter lateral root zone; LR zone, lateral root zone.

We previously investigated the function of *PvRbohA*, which is expressed at higher levels in roots than in other organs [12]. Here, we performed a more detailed analysis to examine *PvRbohA* expression patterns in different root zones of common bean. First, we isolated mRNA from the radicles of 2-day-old wild-type seedlings (Fig. 1A), the inter lateral root (ILR) zone, and LR zone tissues from 4-day-old wild-type seedlings (Fig. 1B). Quantitative RT-PCR analysis revealed significantly higher (∼81%) levels of *PvRbohA* transcript in LR zone tissues compared to the ILR zone (Fig. 1**C**). By contrast, *PvRbohA* transcript levels were significantly lower (∼111%) in radicles with respect to LR zone tissues (Fig. 1**C**). Together, these results indicate that *PvRbohA* is differentially expressed in different zones of wild-type *P. vulgaris* roots; however, its expression increases during LR formation.

## Downregulation of PvRbohA alters root growth in common bean

Next, to investigate whether downregulating *PvRbohA* affects root development in *P. vulgaris*, we generated composite plants with hairy roots expressing the *PvRbohA-RNAi* vector as described in our previous work [14]. We observed root growth parameters at 10 days post transplantation. *PvRbohA-RNAi* lines showed a significant decrease in primary root length (∼22%) and LR density (∼36%) compared to control roots (Fig. 1D-E). As evidenced in our previous studies, silencing of *PvRbohB* significantly affected LR density, whereas ectopic, constitutive expression of *PvRbohB* significantly enhanced LR density, including tertiary and quaternary root number [12,15]. Collectively, these results indicate that *PvRbohA* and *PvRbohB* play distinct roles in the same biological process, i.e., LR formation in *P. vulgaris*.

## The RbohA promoter is active during lateral root emergence

**Figure 2.**
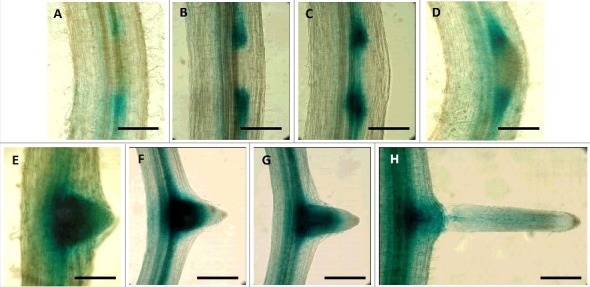
Expression analysis of the *PvRbohA* promoter driving the β-glucuronidase reporter gene in transgenic *P. vulgaris* roots. The spatio-temporal pattern of *PvRbohA* activity was examined in transgenic hairy roots harboring the promoter::GUS-GFP construct incubated in GUS as a substrate. (A-H). Representative images showing promoter activity detected by GUS staining during different stages of LR formation in transgenic roots. (A-B) LR pre-initiation, (C-D) LR primordium initiation, and (E-H) LR emergence. Scale bar, A-E, H: 200 µm; F-G 500 µm. LR: lateral root.

Lateral roots originate deep within the primary root from a small number of founder cells at the periphery of the vascular tissues and must emerge through intervening layers of tissue. LR formation constitutes three major steps: pre-initiation, primordium initiation, and emergence [16]. Great progress has been made in understanding the molecular processes underlying root development in the past decade. Several genes and transcription factors have been characterized that are implicated in LR development, among which *Rboh* genes are especially important. We previously performed detailed analysis of *PvRbohB* promoter activity throughout LR development i.e., from the initial mitotic divisions in the pericycle to LR emergence [13]. Given the importance of the enhanced expression of *PvRbohA* in LR formation zones, we investigated the spatio-temporal expression pattern of *PvRbohA* by examining the expression of a promoter::GUS-GFP construct in transgenic hairy roots of common bean. Microscopy observations revealed that during the LR pre-initiation stage, *PvRbohA* promoter-driven GUS expression occurred in two spots close to the vascular bundles (Fig. 2**A-**B); subsequently, GUS activity increased in the cells flanking the primordium during the LR primordium initiation stage (Fig. 2C-D). At this stage, no GUS expression was observed in the LR primordium. During LR emergence, GUS activity gradually spread to the base of the LR primordium and the vascular bundles (Fig. 2E-G); once the LR emerged from the epidermis, the activity of the *PvRbohA* promoter diminished (Fig. 2H). The relative expression levels of *PvRbohA* during LR formation (Fig. 1C) correlate with the activity of *PvRbohA* promoter (Fig. 2). These observations indicate that the *PvRbohA* promoter is active at all developmental stages of LR formation in *P. vulgaris*.

**Figure 3.**
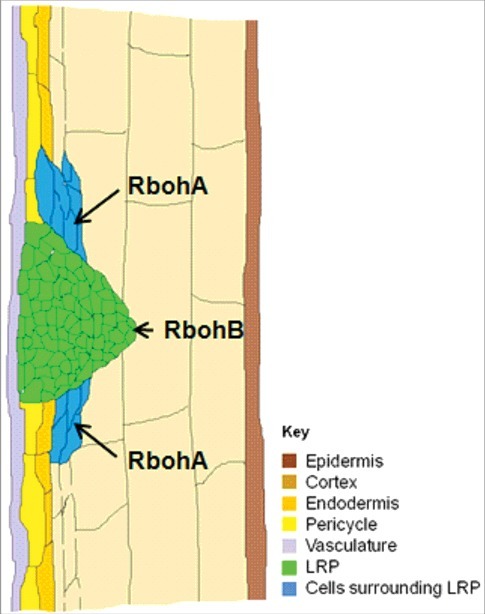
A model of lateral root formation showing a lateral root primordium and the expression of the *PvRbohA* (present study) and *PvRbohB* ^13^ promoters in *P. vulgaris*. LRP, lateral root primordium.

We previously showed that although the *PvRbohB* promoter is active at all LR developmental stages in *P. vulgaris,* its expression is specifically restricted to the LR primordium [13]. Likewise, the *PvRbohA* promoter was also active at all three stages of LR development, but interestingly, its activity varied spatially at each stage. Thus, we propose the following model for LR formation (Fig. 3): During the first two stages of LR formation (LR pre-initiation and LR primordium initiation), the *PvRbohA* promoter is active in cells surrounding the LR primordium, whereas the *PvRbohB* promoter is specifically active in the LR primordium. Nevertheless, both the *PvRbohA* and *PvRbohB* promoters are active during LR emergence. We conclude that both *PvRbohA* and *PvRbohB* contribute to LR formation in *P. vulgaris*.

## Materials and methods

### Root growth analysis

Wild type *Phaseolus vulgaris* seeds were germinated on sterile moistened filter paper in Petri dishes in the dark at 28°C. Radicles (from 2-day-old seedling) and ILR and LR zones of the roots (from 4-day-old seedling) were used to isolate mRNA and measure the levels of *RbohA* transcripts. Composite plants grown in pots containing sterile vermiculite were subjected to root growth parameter measurements. The composite plants were irrigated daily with B&D nutrient medium [17] and maintained in a growth chamber with a 16-h photoperiod and 65% relative humidity at 27 ± 1°C. Transgenic roots expressing red fluorescent protein were selected from individual plants at 10 days post-transplantation, and root growth parameters such as root length and lateral root density were measured. Lateral root density was calculated using the following formula: *D* = LR/*L´*, where *D* = density of lateral roots; LR = number of lateral roots; and *L´* = length of the main root between the first and last lateral root [18].

### Microscopy

Transgenic hairy roots expressing the 35S:*PvRbohA*-*RNAi* vector were selected under an epifluorescence stereomicroscope (SZX7, Olympus, Japan). To analyze the spatio-temporal activity of the promoter, *pPvRbohA*::GUS-GFP transgenic roots were harvested at different time points and stained for GUS activity according to Jefferson [19]. The GUS-stained roots were clarified according to Arthikala et al. [20], and the expression patterns were examined under a light microscope (Axioskop microscope, Zeiss, Germany).
